# Relevant patient characteristics for estimating healthcare needs according to healthcare providers and people with type 2 diabetes: a Delphi survey

**DOI:** 10.1186/s12913-019-4371-z

**Published:** 2019-08-16

**Authors:** Dorijn F. L. Hertroijs, Martijn C. G. J. Brouwers, Arianne M. J. Elissen, Nicolaas C. Schaper, Dirk Ruwaard

**Affiliations:** 1 Department of Health Services Research, CAPHRI Care and Public Health Research Institute, Faculty of Health, Medicine and Life Sciences, Maastricht University, Duboisdomein 30, 6229 GT Maastricht, the Netherlands; 2 Department of Internal Medicine, Division of Endocrinology and Metabolic Diseases, Maastricht University Medical Centre, P. Debyelaan 25, 6229 HX, Maastricht, the Netherlands

**Keywords:** Type 2 diabetes, Delphi panel, Healthcare providers, People with type 2 diabetes, Patient-centered care

## Abstract

**Background:**

Recently, there has been growing interest in providing more tailored, patient-centered care for the treatment of type 2 diabetes mellitus (T2DM). Yet it remains unclear which patient characteristics should be determined to guide such an approach. Therefore, the opinions of healthcare providers (HCP) and people with T2DM about relevant patient characteristics for estimating healthcare needs of people with T2DM were assessed and compared.

**Methods:**

Two separate online Delphi studies were conducted according to the RAND-UCLA Appropriateness Method: one with HCPs (*n* = 22) from Dutch primary and secondary care and one with people with T2DM treated in Dutch primary care (*n* = 46). The relevance of patient characteristics for estimating healthcare needs, defined as the number of yearly consultations, was assessed on a 5-point Likert scale. Characteristics with a median of 4 or 5 and an interquartile range ≤ 1.5 were considered relevant with consensus. Participants were also asked to select the top 5 of most relevant patient characteristics. To determine the overall top 5, the mean relative importance score of each characteristic was calculated.

**Results:**

In two Delphi rounds, 28 and 15 patient characteristics were rated by HCPs and people with T2DM, respectively. Both HCPs and people with T2DM found health-related characteristics relevant for estimating healthcare needs of people with T2DM. However, HCPs preferred to estimate healthcare needs using person- and context-related characteristics. They ranked self-efficacy as the most relevant estimator. In contrast, people with T2DM were more in favor of health-related characteristics and ranked HbA1c as the most relevant estimator.

**Conclusions:**

The findings show that there is discrepancy in opinions on relevant patient characteristics for estimating healthcare needs between HCPs and people with T2DM. To achieve more tailored, patient-centered care, it is important that both groups agree on the topics to be discussed during patient consultations.

**Electronic supplementary material:**

The online version of this article (10.1186/s12913-019-4371-z) contains supplementary material, which is available to authorized users.

## Background

Type 2 diabetes mellitus (T2DM) is one of the most prevalent chronic conditions and a worldwide public health priority [1, 2]. In Europe, an estimated 59.8 million individuals suffer from T2DM. This number is expected to rise to 71.1 million by the year 2040, largely due to the aging of Europe’s population [1, 2]. People with T2DM are at high risk for developing complications, such as cardiovascular disease and kidney failure, which in turn lead to increased healthcare costs [2, 3]. Maintaining a good glycemic, blood pressure, and lipid control could prevent these complications [4, 5].

A large proportion of T2DM care is based on self-management, which is defined as the active participation of people with T2DM in their treatment [6]. In accordance with evidence-based care protocols for T2DM treatment, people with T2DM regularly visit healthcare providers (HCP) who should assist them in obtaining the knowledge and skills to self-manage their disease with confidence (e.g. day-to-day blood glucose monitoring, medication intake and lifestyle adjustment) [7, 8]. Adhering to these behaviors has been positively correlated with glycemic control [9, 10]. However, the guidelines for T2DM treatment are usually highly standardized, resulting in differential treatment effects [11, 12]. This indicates a need for more patient-centered care, in which patient characteristics are used to predict the healthcare needs of people with T2DM and to adjust care, including self-management education and support, accordingly. Recently, there has been growing interest in providing patient-centered care for the treatment of T2DM [13–15]. Thus far, it is unclear which patient characteristics should be identified to implement such an approach. Several studies have pointed towards psychosocial characteristics, such as self-esteem, self-efficacy and quality of life to tailor care [16, 17], whereas others emphasize the relevance of biomedical characteristics, such as body mass index (BMI) and HbA1c [18, 19].

As a first step towards more patient-centered care for people with T2DM, the Dutch PROFILe (PROFiling people with type 2 diabetes healthcare needs to support Integrated, person-centered models for Long-term disease management) project started in 2014. PROFILe aims to develop, validate and test so-called ‘patient profiles’ as an instrument for tailored T2DM management in practice [20]. Based on the assessment of patient characteristics, people with T2DM with similar healthcare needs, preferences and abilities can be stratified into the appropriate profile, for which optimal combinations of professional-led care and self-management support can be developed. To identify relevant patient- and disease individual characteristics a systematic literature review was conducted [21] and the associations of 38 of such characteristics with HbA1c were analyzed using cross-sectional data of people with T2DM [17]. Furthermore, the electronic health records of people with T2DM were used to identify latent glycemic control trajectories, which are unobserved trajectories that capture the glycemic control of individuals, and to build a model that predicts these trajectories using patient- and disease individual characteristics [22]. Another, more qualitative approach is to gain insight into the opinions of HCPs and people with T2DM regarding this subject. To achieve true translational research, it is important to include the voices of HCPs and people with T2DM in research due to their experiential knowledge [23]. Therefore and within the context of the PROFILe project, the objective of this study was to assess and compare the opinions of HCPs and people with T2DM about relevant patient characteristics for estimating healthcare needs in primary care.

## Methods

### Participants

Two separate Delphi studies were conducted: one with HCPs and one with people with T2DM.

The first Delphi study was conducted from September through October 2016 and included a purposive and representative sample of HCPs (general practitioners, practice nurses [who support the general practitioner in primary care], specially trained diabetes nurses, dieticians, internists, psychologists and pharmacists) recommended in the care protocols to be part of the multidisciplinary care team for the treatment of T2DM. The authors composed a list of HCPs (*n* = 20) from their own network who treat or used to treat people with T2DM in the Dutch healthcare system and/or have extensive knowledge on the organization of T2DM care in the Netherlands. These HCPs were asked to participate and to recommend colleagues (*n* = 6) who might be willing to participate as well. Furthermore, the Dutch Professional Association of T2DM Care Providers (EADV) and the Dutch Dietician Nutrition Organization (DNO) were contacted for recommendations on HCPs interested in participation (*n* = 8). In total, 34 HCPs were invited to participate and give written informed consent.

The second Delphi study focused on people with T2DM with a diagnosis of T2DM and took place between June and August 2017. For the recruitment of people with T2DM, we contacted one general practitioner with a practice in the north of the Netherlands in which 109 people with T2DM were treated. People with T2DM who also had a diagnosis of dementia were excluded from participation, all other people with T2DM were asked if they were willing to participate and give written informed consent.

### Procedure

Both Delphi studies were conducted according to the RAND/UCLA appropriateness method and consisted of two rounds [24].

#### First round

In the first round, participants (i.e. both HCPs and people with T2DM) received a survey which consisted of questions rating the relevance of patient characteristics for estimating the healthcare needs of people with T2DM on a Likert scale ranging from 1 (totally irrelevant) to 5 (extremely relevant) (Additional file 1). Healthcare needs was defined as the number of yearly consultations needed with a general practitioner and/or practice nurse. Besides rating each characteristic, participants were asked for their opinion on why they considered certain patient characteristics to be more or less relevant for estimating healthcare needs. They were also asked to select the top 5 of most relevant patient characteristics for estimating healthcare needs and to report other characteristics that they found relevant, but were not included in the survey. A questionnaire on demographic characteristics of the participants was also included.

#### Second round

In the second round, participants received a summary of the results of all partaking individuals in the first round. This allowed them to re-assess their original opinion about the level of relevance of characteristics on which no consensus was reached between participants.

Next, participants were asked to rate the importance of the characteristics with no consensus and, if any, of the characteristics that were added by the participants in the first round. They were again asked to report the top 5 of most important characteristics for estimating healthcare needs.

### Characteristics

#### Healthcare provider survey

The healthcare provider survey of the first round was composed of 18 characteristics that were found to be associated with or able to predict glycemic control in previously conducted empirical research [17, 21, 22]. To structure these characteristics, they were divided into the three categories of the Anderson and Newman model assumed to be predictors of health services use: person-, context- and health-related patient characteristics [25]. In the person-related category, age, sex and self-efficacy were included. Two context-related characteristics were analyzed: income and educational level. Characteristics included in the health-related category were: HbA1c, systolic blood pressure, LDL-cholesterol, triglycerides, BMI, cardiovascular disease, nephropathy, retinopathy, neuropathy, T2DM duration, T2DM medication, diabetes-related distress and quality of life. The HCP survey of the second round included characteristics of which no consensus was reached in the first round and characteristics that were added by the HCPs in the first round, if any.

#### Patient survey

To improve understandability we included similar, but fewer characteristics in the patient survey of the first round compared to the healthcare provider survey. Except for HbA1c and BMI (which was named ‘weight’ in the patient survey), all other health-related characteristics were excluded from the survey, because we felt that not all people with T2DM would be able to understand the meaning of these characteristics. The T2DM-related complications nephropathy, retinopathy, neuropathy and cardiovascular disease were simplified by summarizing them in one characteristic called ‘having other diseases’. In addition, we added the top 5 of most relevant characteristics for estimating healthcare needs as rated by HCPs to the patient survey, but only if we felt people with T2DM would understand the meaning of these characteristics. In total, the patient survey in the first round consisted of 13 characteristics. The patient survey of the second round included characteristics of which no consensus was reached in the first round and characteristics that were added by the people with T2DM in the first round, if any.

### Statistical analyses

Descriptive analyses were conducted to assess the demographic characteristics of the participants. The relevance of the person-, context-, and health-related characteristics for the questions with a 5-point Likert scale was classified into three categories based on median scores: not relevant (median 1–2), uncertain (median 3) and relevant (median 4–5). To determine the level of consensus between participants, the interquartile range (IQR) was calculated for each characteristic. An IQR ≤ 1.5 was considered as consensus, meaning that at least 50% of all ratings are situated within 1.5 points around the median rating of the participants [26]. Characteristics with a median of 3 and/or an IQR > 1.5 in the first round were considered not relevant and presented again in the second round.

To determine the overall top 5 of most relevant characteristics for estimating healthcare needs of both Delphi studies, each characteristic was awarded points based on the top 5 placement of each individual. A characteristic that was considered as most relevant by an individual received 5 points, the second most relevant characteristic 4 points, etc. The mean relative importance score of each characteristic was assessed by dividing the total awarded points for each characteristic by the total number of participants included in each Delphi study. Significant differences (*p* < 0.05) between the mean relative importance scores of the characteristics in the HCP and patient top 5 s were determined using two-sample t-tests.

All analyses were performed using R Studio version 1.0.153.

## Results

First the results of the Delphi study with HCPs are given, followed by the results of the Delphi study with people with T2DM and finally the outcomes of both Delphi studies are compared.

### Healthcare providers

#### Demographic characteristics HCPs

In total, 23 of the 34 (67.6%) invited HCPs agreed to participate. One healthcare provider did not complete the first survey round and was therefore excluded; twenty-two HCPs completed all Delphi rounds. Demographic characteristics of the HCPs are shown in Table 1. Mean age was 51.4 years (SD 9.5), 14 HCPs (63.6%) were female and the median period of professional experience was 15 years (range 1–35).

**Table 1:** Characteristics of healthcare providers who responded to the survey (*n* = 22)

Characteristic	N
Sex *n* (%)	
Female	14 (63.6)
Male	8 (36.3)
Age, mean (sd)	51.4 (9.5)
Profession *n* (%)	
General practitioner	4 (18.1)
Practice nurse	4 (18.1)
Diabetes nurse	3 (13.6)
Dietician	6 (27.3)
Internist	3 (13.6)
Psychologist	1 (4.5)
Pharmacist	1 (4.5)
Professional experience in diabetes care, median number of years (range)	15 (1–35)
Work setting *n* (%)	
Primary care	14 (63.6)
Hospital	5 (22.7)
Primary care and hospital	1 (4.5)
Other	2 (9.1)

#### Delphi rounds 1 and 2 healthcare providers

The results of round 1 in the HCPs are shown in Table 2, 18 characteristics were rated as relevant. Of these, 15 characteristics were considered relevant with consensus (median ≥ 4, IQR ≤1.5) for estimating healthcare needs. The highest ratings of relevance were observed for self-efficacy and nephropathy. Consensus between participants was not reached for the three characteristics: sex, income and triglycerides. Therefore, these characteristics were presented again in the second Delphi round. There were no characteristics considered irrelevant with consensus. HCPs added the characteristics social support (*n* = 7) (e.g. family relations and living situation), comorbidities (*n* = 4), cultural background (*n* = 3), lifestyle (*n* = 2), profession (*n* = 2), language barrier (*n* = 2), ‘taking responsibility for disease’ (e.g. taking medications and following a healthy diet) (*n* = 2), financial situation (*n* = 1), psychological characteristics (n = 1) and emotional characteristics (*n* = 1). These were included in the HCP survey of the second round.

**Table 2:** Results of Delphi round 1 and round 2 for healthcare providers

	Round 1		Round 2	
	Median	IQR	Median	IQR
Person-related characteristics				
Age	4	0		
Sex	3	1	2	1
Self-efficacy	5	1		
Lifestyle			5	1
Taking responsibility for disease			5	1
Context-related characteristics				
Educational level	4	1		
Income	3	1		
Social support			4	1
Cultural background			4	1
Profession			3	1
Financial situation			4	1
Language barrier			4	0
Health-related characteristics				
Quality of life	4	1		
HbA1c	4	0		
Systolic blood pressure	4	0		
LDL-cholesterol	4	1		
Triglycerides	3	1.75	2	1
Body mass index	4	1		
Cardiovascular disease	4	1		
Nephropathy	5	1		
Retinopathy	4	1		
Neuropathy	4	1		
Diabetes duration	4	1		
Diabetes medication	4	0		
Diabetes related distress	4	0		
Co-morbidity			4	1
Psychological characteristics			4	0
Emotional characteristics			4	0

Relevance of characteristics: median 1–2 = not relevant, median 3 = uncertain and median 4–5 = relevant

Consensus: IQR ≤ 1.5 = consensus, IQR > 1.5 = no consensus

Characteristics with a median of 3 and/or IQR > 1.5 in the first round, were presented again in the Delphi survey second round

Characteristics that were added by HCPs in the first round were presented in the Delphi survey in the second round

In the second round characteristics with no consensus in the first round (n = 3) were re-assessed and the characteristics added by HCPs were rated for the first time (Table 2). HCPs reached consensus on the characteristics sex and triglycerides, which they found irrelevant for estimating healthcare needs. Consensus was also reached for income, which they found not relevant for estimating healthcare needs. All characteristics that were added by HCPs were considered relevant with consensus, except for the characteristic profession for which the relevance was found uncertain. Both rounds combined, HCPs rated a total of 28 characteristics.

The top 5 of most relevant patient characteristics according to HCPs consisted of: lifestyle, ‘taking responsibility of disease’ and social support (context-related characteristics) as well as self-efficacy and health-related characteristic quality of life (person-related characteristics).

### People with T2DM

#### Demographic characteristics people with T2DM

A hundred people with T2DM were invited to participate in the study, of whom 48 agreed (48%). People with T2DM who did not agree to participate had a significantly shorter average T2DM duration compared with people with T2DM who did agree to participate (7.9 vs. 11.7 years, 95% CI: − 7.04 - -0.44, *p*-value = 0.027). Other characteristics did not differ. The first Delphi round was completed by 46 people with T2DM and the second round by 41 people with T2DM. Mean age was 68.8 years (SD 9.9), 25 (54.3%) people with T2DM were female (Table 3).

**Table 3:** Characteristics of people with T2DM who responded to the survey (*n* = 46)

Characteristic	
Sex *n* (%)	
Female	25 (54.3)
Male	21 (45.7)
Age, mean (sd)	68.8 (9.9)
Country of birth *n* (%)	
Netherlands	45 (97.8)
Other	1 (2.2)
Educational level *n* (%)^a^	
Higher professional education	9 (20.5)
Middle professional education	7 (15.9)
High School	21 (47.7)
Elementary school/no education	7 (15.9)
Not recorded	2
Diabetes duration, mean years (sd)	11.7 (9.6)
Diabetes medication *n* (%)	
None	11 (23.9)
Glucose-lowering drugs only	26 (56.2)
Insulin only	2 (4.3)
Glucose-lowering drugs and insulin	7 (15.2)

^a^percentages are out of total with recorded values

#### Delphi rounds 1 and 2 people with T2DM

As previously described, similar, but fewer characteristics were included in the patient survey compared with the healthcare provider survey. In addition, we added the top 5 of most relevant characteristics for estimating healthcare needs as rated by HCPs to the patient survey, except for lifestyle and ‘taking responsibility for disease’, because we felt people with T2DM would confuse these with weight and self-efficacy, respectively. In total, people with T2DM rated 13 characteristics in the first Delphi round (Table 4). Eight characteristics were considered relevant with consensus. Consensus between people with T2DM was not reached about the relevance of age, sex, income and social support. Therefore, these characteristics were presented again in the second Delphi round. Educational level was considered irrelevant with consensus for estimating healthcare needs. People with T2DM added the characteristics genetics and insecurity/fear to the final Delphi round.

**Table 4:** Results of Delphi rounds 1 and 2 for people with T2DM

	Round 1		Round 2	
	Median	IQR	Median	IQR
Person-related characteristics				
Age	2	2	3	2
Sex	2	1.75	2	1
Self-efficacy	4	1		
Context-related characteristics				
Educational level	2	1		
Income	2	2	2	2
Social support	3	2	3	2
Health-related characteristics				
Quality of life	4	1		
HbA1c	4	0		
Weight	4	0.75		
Diabetes duration	4	1		
Diabetes medication	4	1		
Diabetes related distress	4	1		
Comorbidity	4	1		
Genetics			4	2
Insecurity/fear			3	2

Relevance of characteristics: median 1–2 = not relevant, median 3 = uncertain and median 4–5 = relevant

Consensus: IQR ≤ 1.5 = consensus, IQR > 1.5 = no consensus

Characteristics with a median of 3 and/or IQR > 1.5 in the first round, were presented again in the Delphi survey second round

Characteristics that were added by HCPs in the first round were presented in the Delphi survey in the second round

People with T2DM rated six characteristics in the second Delphi round. Sex was considered irrelevant with consensus. No consensus was reached on the remaining five characteristics (Table 4). Both rounds combined, people with T2DM rated a total of 15 characteristics.

The top 5 of most relevant patient characteristics according to people with T2DM consisted of: HbA1c, T2DM medication, quality of life and co-morbidities (health-related characteristics) as well as self-efficacy (person-related characteristic).

### Comparison between HCPs and people with T2DM

Of the total set (*n* = 30) of unique characteristics included across the two surveys, 28 were rated by HCPs and 15 by people with T2DM. Out of all these characteristics, 13 were rated by both the HCP and the people with T2DM. In both groups, eight of these characteristics achieved consensus for relevance, including all health-related characteristics. Both groups agreed that sex was irrelevant for estimating healthcare needs. There were also some discrepancies between HCPs and people with T2DM on person- and context-related characteristics. HCPs found age, educational level and social support relevant with consensus, but people with T2DM found educational level irrelevant with consensus and were uncertain about the usage of age and social support to estimate healthcare needs.

**Fig. 1:**
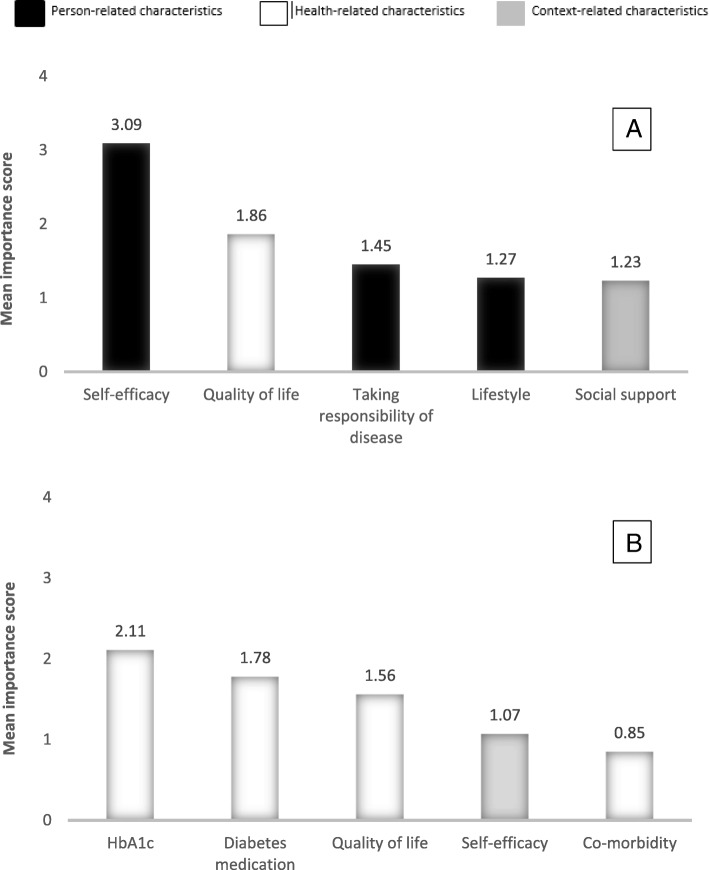
Most relevant 5 characteristics for estimating healthcare needs according to HCPs (**a**) and people with type 2 diabetes (**b**)

Figure 1 shows the mean relative importance scores of the five most relevant characteristics for estimating healthcare needs according to HCPs (A) and people with T2DM (B). The top 5 according to HCPs mainly consisted of person- and context-related characteristics, whereas for people with T2DM the top 5 mainly consisted of health-related characteristics. HCPs rated self-efficacy as the most relevant patient characteristic with a mean relative importance score of 3.09, whereas people with T2DM rated HbA1c as the most relevant characteristic with a relative mean importance score of 2.11. The two characteristics that were present in both the HCP and patients top 5 s, were self-efficacy and quality of life. Significant differences between the mean relative important scores were found for all characteristics in both top 5 s that were rated by both HCPs and patients, except for quality of life (*p* = 0.573) and comorbidity (*p* = 0.344).

## Discussion

### Principal findings

In the present study, HCPs and people with T2DM were asked to give their opinion about the relevance of patient characteristics for estimating healthcare needs of people with T2DM. In two Delphi rounds, 28 and 15 patient characteristics were rated by HCPs and people with T2DM, respectively. Except for triglycerides, genetics and insecurity/fear, all health-related characteristics were found to be relevant with consensus for estimating healthcare needs by both HCPs and people with T2DM.

Discrepancies in opinions between HCPs and people with T2DM were observed for person- and context-related characteristics. HCPs found 75% of these characteristics relevant for estimating healthcare needs, whereas people with T2DM only found 17% relevant. A striking discrepancy was also seen in the top 5 of most relevant patient characteristics for estimating healthcare needs between HCPs and people with T2DM. The top 5 of HCPs mostly consisted of person- and context-related characteristics and they thought that self-efficacy was most relevant for estimating healthcare needs. In contrast, the top 5 of people with T2DM mostly consisted of health-related characteristics and they ranked HbA1c as the most relevant estimator.

### Comparison with other studies

Previous research has suggested that more emphasis should be placed on person- and context-related characteristics in the treatment of T2DM [27, 28]. In the current study, self-efficacy for example, defined as an individual’s confidence in being able to carry out a behavior, was rated as the most and fourth most relevant characteristic for estimating healthcare needs by HCPs and patients, respectively, and has been associated with lower HbA1c levels and T2DM management- and problem solving behavior [29, 30]. A healthcare provider’s knowledge on the self-efficacy of people with T2DM and other person- and context-related characteristics could enhance self-management education and support and the development of mutually accepted treatment goals, referred to as shared decision making (SDM) [28, 31–34]. Similar to the outcome of the current study, most HCPs agree on such a broad, whole person approach to the treatment of T2DM [35, 36]. However, from the current study it remains unclear whether HCPs practice such an approach. Previous research has shown that HCPs often lack the time, skills and resources to provide self-management education and support and SDM is not yet embedded in clinical practice [37]. Instead, patient consultations with a healthcare provider seem to focus on clinically orientated issues, such as optimal blood glucose levels [31]. Past qualitative research has shown that people with T2DM were unable to describe the role of the practice nurse beyond clinical checks [38]. They also were not sure what else they could expect from their practice nurse. On the other hand, it could be that HCPs do discuss person- and context-related characteristics during patient consultations, but people with T2DM might be unaware of this or not open to it. These arguments might explain why people with T2DM considered HbA1c as the most relevant characteristic for estimating the healthcare needs of people with T2DM in the current study.

### Strengths and limitations

A strength of this study was the unique inclusion of HCPs as well as people with T2DM. A Delphi panel is often referred to as an ‘expert panel’ and assumed to include professionally and scientifically qualified participants [39]. People with T2DM do not fall under this category. We did, however, decide to include them, because of their relevant knowledge and experience on the topic and because knowing the opinions of both groups can improve the development of patient-centered care. On the other hand, the Delphi method is context free, which could explain the differences in opinion between HCPs and people with T2DM. We do not know for example, if people with T2DM were trying to see things from the HCPs’ perspective, despite the provided instructions that asked them to make their own judgement. In that case, a true qualitative method would have been better to elicit people with T2DM’ views.

Given the scale of this study, we decided to only select patients from one primary care practice. This does mean that the included patients all live in the same region and are treated by the same HCPs, which makes it difficult to generalize the results to people with T2DM in other countries and other regions Dutch regions. However, since Dutch general practitioners and especially practice nurses (who treat more patients with diabetes than the GP) strictly adhere to the guidelines for T2DM treatment [40], it is likely that the included patients received T2DM care similar to the care of patients from other primary care practices. Moreover, as patients within the practice differ in terms of which HCP they most frequently see for their T2DM – there is one GP and three practice nurses providing T2DM care – we expect the influence of provider attitude and interpersonal style on patients’ opinions to be limited. The included HCPs formed a multidisciplinary Delphi panel. In the Netherlands multidisciplinary cooperation within T2DM teams – comprising not only general practitioners and practice nurses, but also T2DM nurses, dieticians, psychologists and, to a limited extent, internists – forms an important part of T2DM care [41]. They refer people with T2DM to each other and mutually discuss treatment plans. The diversity of our panelists represented the range of HCPs that are involved in the treatment of people with T2DM and their opinions. Only Dutch HCPs were included. We tried to arrange face-to-face meetings with the participants, to allow for more in-depth discussion about the ratings and investigate areas of disagreement. Due to time-constraints of the participants, the decision was made to conduct an online Delphi survey instead. To gain more understanding of the ratings, we did include open questions. Finally, the patient characteristics that were included in the Delphi surveys were derived from studies that were previously conducted as part of the PROFILe project, which included an in-depth systematic literature search [21]. It is, however, possible that we missed relevant patient characteristics for estimating healthcare needs of people with T2DM. Research has, for example, suggested that environmental factors, such as social stratification and political context, have an impact on people’s health [28, 42]. These factors are, however, difficult for HCPs and people with T2DM to influence, and were therefore not included in the surveys. Furthermore, participants were given the chance to provide a list of patient characteristics that they found relevant for estimating healthcare needs of people with T2DM and were not included in the survey of the first round.

### Clinical implications

The findings of this study complement the results derived from previous empirical research on relevant patient characteristics for estimating healthcare needs. They are important for both HCPs involved in the treatment of people with T2DM and researchers focusing on the development of patient-centered care. The findings suggest that there is discrepancy in opinions on relevant patient characteristics for estimating healthcare needs between HCPs and people with T2DM. To improve SDM and encourage patient-centered care, it is important that both groups agree on what topics should be discussed during patient consultations. People with T2DM have previously reported that they would like their healthcare provider to show more interest in their life and provide more explanation and involvement in T2DM management, such as providing lifestyle advice and discussing treatment options [31, 38]. Indeed, a recent study on the implementation of a structured T2DM consultation model with a focus on person- and context-related patient characteristics, led to an increase in patient involvement and a substantial number of satisfied people with T2DM [43]. In the current study, HCPs and people with T2DM agreed that self-efficacy and quality of life are relevant patient characteristics for estimating healthcare needs. The measurement of these characteristics should therefore to be included in routine care, for example as part of the intake of people with newly diagnosed T2DM. To save time, people with T2DM could fill in questionnaires that measure these characteristics before their visit with a HCP. Identifying self-efficacy and quality of life in diabetes management allows HCPs to know which aspects of the lives of people with T2DM are most important and which activities they are facing most difficulties with [44]. This has important implications on targeting person-centered education interventions.

## Conclusions

Both HCPs and patients reported health-related characteristics as relevant for estimating patients’ healthcare needs. However, the findings also showed a discrepancy in opinions between HCPs and patients. HCPs found context-related and person-related characteristics more relevant to estimate healthcare needs than patients did. They ranked self-efficacy as the most important estimator. In contrast, patient found health-related characteristics more relevant and ranked HbA1c as the most relevant estimator. To achieve more tailored, patient-centered care, it is important that both groups agree on the topics to be discussed during patient consultations. Future research should focus on improving the skills and tools HCPs need to take into account patient’s person- and context-related characteristics and gaining more understanding on the preferences of people with T2DM regarding diabetes care. In the next step of the PROFILe project, a discrete choice experiment will be conducted to elicit preferences of people with T2DM for each of the identified latent glycemic control trajectories [20]. In combination with a consultation model, where person- and context-related characteristics will be discussed, this will enable HCPs to provide patient-centered care by taking into account people with T2DM’ care preferences, abilities, and needs.

## Additional file

Additional file 1: Delphi panel questionnaires for healthcare providers and patients. (PDF 774 kb)

## Data Availability

The anonymized dataset necessary to replicate this study’s findings will be available upon request to the corresponding author.

## Abbreviations

BMI: Body mass index
DNO: Dutch Dietician Nutrition Organization
EADV: Dutch Professional Association of T2DM Care Providers
HCP: Healthcare provider
PROFILe: PROFiling people with T2DM’ healthcare needs to support Integrated, person-centered models for Long-term disease management
T2DM: Type 2 T2DM
