# Supplementary material for: Relevant patient characteristics for estimating healthcare needs according to healthcare providers and people with type 2 diabetes: a Delphi survey
Source: BMC Health Serv Res. 2019 Aug 16;19:575. doi: 10.1186/s12913-019-4371-z (PMC6698036; doi:10.1186/s12913-019-4371-z)
Supplement: Supplementary file 1 — Delphi panel questionnaires for healthcare providers and patients. (PDF 774 kb) [file 12913_2019_4371_MOESM1_ESM.pdf]

Questionnaire Delphi panel healthcare providers

1. What is your age?

2. What is your gender?
   - ☐ Man
   - ☐ Woman

3. What is your profession?
   - ☐ Pharmacist
   - ☐ Diabetes nurse
   - ☐ Dietician
   - ☐ General practitioner
   - ☐ Internist
   - ☐ Practice nurse
   - ☐ Psychologist

4. Where do you work?
   - ☐ Primary care
   - ☐ Secondary care
   - ☐ Other,

5. How many years of experience do you have treating patients with type 2 diabetes?

6. You treat multiple patients. Most likely not all of these patients have type 2 diabetes. What percentage of the total number of patients you treat has type 2 diabetes?

The next questions are about relevant patient characteristics for estimating healthcare needs of patients with type 2 diabetes. The healthcare needs are subdivided into person-related and health-related characteristics.

Maastricht University Maastricht UMC+

7. How relevant do you think these person-related patient characteristics are for estimating healthcare needs of patients with type 2 diabetes?

**Characteristics**	Not at all relevant	Not relevant	Neutral	Relevant	Very relevant
Age	☐	☐	☐	☐	☐
Sex	☐	☐	☐	☐	☐
Educational level	☐	☐	☐	☐	☐
Self-efficacy	☐	☐	☐	☐	☐
Income	☐	☐	☐	☐	☐
Quality of life	☐	☐	☐	☐	☐

8. Could you clarify for at least 3 person-related patient characteristics from the previous question why you find them relevant or not?

9. How relevant do you think these health-related patient characteristics are for estimating healthcare needs of patients with type 2 diabetes?

**Characteristics**	Not at all relevant	Not relevant	Neutral	Relevant	Very relevant
HbA1c	☐	☐	☐	☐	☐
Systolic blood pressure	☐	☐	☐	☐	☐
LDL-cholesterol	☐	☐	☐	☐	☐
Body Mass Index (BMI)	☐	☐	☐	☐	☐

Cardiovascular disease	☐	☐	☐	☐	☐
Nephropathy	☐	☐	☐	☐	☐
Neuropathy	☐	☐	☐	☐	☐
Diabetes duration	☐	☐	☐	☐	☐
Glucose lowering drugs	☐	☐	☐	☐	☐
Diabetes related distress	☐	☐	☐	☐	☐

10. Could you clarify for at least 5 health-related characteristics from the previous question why you find them relevant or not?

11. Are there other characteristics that were not named, but that you find relevant for estimating the healthcare needs of patients with type 2 diabetes?
    - ☐ Yes *(continue to question 12)*
    - ☐ Nee *(continue to question 13)*

12. Which other characteristics, according to you, are relevant for estimating the healthcare needs of patients with type 2 diabetes?

13. Below you can add your top 5 of the previously mentioned characteristics that you found relevant. Add a 1 to the characteristic that you find most relevant, a 2 to the characteristic that you find most relevant thereafter, etc. You may also add characteristics that were not previously mentioned, but that you do find relevant.

- ☐ age
- ☐ Sex
- ☐ Educational level
- ☐ Quality of life
- ☐ Income
- ☐ Self-efficacy
- ☐ HbA1c
- ☐ Systolic blood pressure
- ☐ LDL-cholesterol
- ☐ Triglycerides
- ☐ Body Mass Index
- ☐ Cardiovascular disease
- ☐ Nephropathy
- ☐ Diabetes duration
- ☐ Glucose lowering drugs
- ☐ Diabetes-related distress
- ☐ Other, ____________
- ☐ Other, ____________

14. Could you please explain your top 5?

Thank you very much for filling in the questionnaire.

Questionnaire Delphi panel patients

15. What is your gender?

- ☐ Man
- ☐ Woman

16. What is your highest level of school completed?

- ☐ None/elementary school
- ☐ VMBO/MAVO/LBO
- ☐ MBO (MTS, MEAO)
- ☐ HAVO/VWO (HBS, MMS)
- ☐ HBO/WO (HTS, HEAO)

17. Wat is your birth country?

- ☐ the Netherlands
- ☐ Surinam
- ☐ Netherlands Antilles
- ☐ Aruba
- ☐ Turkey
- ☐ Marocco
- ☐ Other, [ ]

18. In what year were you diagnosed with type 2 diabetes? [ ]

19. What glucose lowering drug(s) do you use?

- ☐ None
- ☐ Oral
- ☐ Insulin
- ☐ Oral and insulin

The next questions are about which characteristics your find relevant for estimating how many contact moments you need with your general practitioner, practice nurse or diabetes nurse.

Maastricht University Maastricht UMC+

<u>**We start with an example. You do not have to answer this example.**</u>

Example:

State for the characteristic below (hair colour) how relevant you think this characteristic is for estimating the number of contact moments you need with your general practitioner, practice nurse or diabetes nurse:

	Not at all relevant	Not relevant	Neutral	Relevant	Very relevant
Hair colour	☐	☐	☐	☐	☐

Imagine you have red hair. You think that because of your red hair, you need more contact moments with your general practitioner, practice nurse or diabetes nurse. Therefore, you find hair color relevant or very relevant and you add a cross under 'relevant' or 'very relevant':

	Not at all relevant	Not relevant	Neutral	Relevant	Very relevant
Hair colour	☐	☐	☐	☐	√

You might think that you need the exact same number of contact moments with your general practitioner, practice nurse or diabetes nurse as people with another hair colour. In that case, you add a cross under 'not relevant' or 'not at all relevant':

	Not at all relevant	Not relevant	Neutral	Relevant	Very relevant
Hair colour	☐	√	☐	☐	☐

This was the example. On the next page questions are asked about the characteristics that are important for the study.

20. State for each characteristic how relevant you think the characteristic is for estimating the number of contact moments with your general practitioner, practice nurse of diabetes nurse:

**Characteristics**	Not at all relevant	Not relevant	Neutral	Relevant	Very relevant
Age	☐	☐	☐	☐	☐
Sex	☐	☐	☐	☐	☐
Educational level	☐	☐	☐	☐	☐
Quality of life	☐	☐	☐	☐	☐
Income	☐	☐	☐	☐	☐
Self-efficacy	☐	☐	☐	☐	☐
Blood sugar	☐	☐	☐	☐	☐
Weight	☐	☐	☐	☐	☐
Diabetes duration	☐	☐	☐	☐	☐
Stress	☐	☐	☐	☐	☐
Social support	☐	☐	☐	☐	☐
Diabetes medication	☐	☐	☐	☐	☐
Having other diseases	☐	☐	☐	☐	☐

21. Could you clarify for a few characteristics why you find them relevant or not?

8. Are there other characteristics that were not named, but that you find relevant for estimating the number of contact moments with your general practitioner, practice nurse or diabetes nurse?
- ☐ Yes *(continue to question 9)*
- ☐ Nee *(continue to question 10)*

9. Which characteristics, according to you, are relevant for estimating the number of contact moments with your general practitioner, practice nurse or diabetes nurse?

10. Below you can add your <u>top 5</u> of the previously mentioned characteristics that you found relevant. Add a 1 to the characteristic that you find most relevant, a 2 to the characteristic that you find most relevant thereafter, etc. You may also add characteristics that were not previously mentioned, but that you do find relevant.

☐ age	☐ Sex	☐ Educational level
☐ Quality of life	☐ Income	☐ Self efficacy
☐ Blood sugar	☐ Weight	☐ Having other diseases
☐ Stress	☐ Social support	☐ Diabetes duration
☐ Diabetes medication		

☐ Other, ________________

☐ Other, ________________

11. Could you please explain your top 5?

Thank you very much for filling in the questionnaire.
